# Who First Suggested Headed Pins for Artificial Teeth?

**Published:** 1862-07

**Authors:** 


### WHO FIRST SUGGESTED HEADED PINS FOR ARTIFICIAL TEETH?

HILLSBORO, Ill., June 23, 1862.

DR. WATT,—*Dear Sir :*—In May, 1861, I sent a letter to Dr. W. B. Franklin, of New York, describing, as clearly as I could, an improvement in making pins for artificial teeth, and urging him to consider the matter, and if the teeth could be made with such pins, to take immediate steps to have them manufactured for the good of the profession, not caring for any other reward than the consciousness of doing a *little* for so noble a calling as dentistry. Dr. Franklin, in his reply to my letter, stated two obstacles that presented themselves to his mind in making teeth on the plan proposed. He promised, however, to lay the matter before the tooth-makers, and expressed a hope that the difficulty he thought of might be overcome. Imagine my surprise, when, looking at the *November* issue of the "Vulcanite," under head "Platina Pins in Teeth," to see the very same improvement in pins, and almost the very same description, sent in my letter the previous May, closing the notice with a eulogy to Dr. S. S. White, giving him the whole credit for this move in the right direction. You will observe that, between May, 1861, and September, 1861, there was an issue of the "Vulcanite," and no mention made of the suggestion contained in my letter.

Dr. Franklin never stated whether or not he placed my letter before the tooth-makers, as he promised. He did state that, in the following August, he exhibited, in behalf of Dr. S. S. White, samples of artificial teeth made with headed pins, at the Dental Convention at New Haven, Conn.

It is very strange, as he remarks, with this remarkable hint before him, that he did not remember my letter describing the very improvement he was then showing to the Convention.

I regard headed pins for vulcanite work all that can be desired; and as Dr. S. S. White derives all the pecuniary gains, I think it but just and right that the one who first made public so important and useful an improvement be known and placed in the proper relation with this matter. Dr. Franklin says Dr. S. S. White was probably getting up models for making headed pins previous to my letter of May, 1861. That may be all true ; but, at the same time, the idea had suggested itself to my mind months before I wrote to him, and I had frequently conversed with my partner concerning it.

NEW METHOD OF FASTENING ARTIFICIAL TEETH TO PLATES.

I sent, at the same time, a description and model, which I herewith enclose, to the editor of "Vulcanite," requesting him to publish the same, which letter brought the block of artificial teeth you will find, with the model, stating that the parties who handed him this sample were making application for a patent.

The editor thought this "sample" embodied the idea involved in my model, (which was the same as this one I send you) and took no farther notice of it.

It occurs to me that this is a very unfair conclusion, as you will see by comparing the two. Dr. Franklin stated that the patent was contingent upon the small hole extending lengthwise of the block. The principle, and security of attachment to the plate, relied on in my model, is the continuous groove of hard rubber, and standards of the same, let down from the base of groove into the heel of the tooth. The large part of groove looking towards the points of teeth will render the attachment very firm and easily accomplished.

The varnished part of model represents the gums. The shoulder marked on the labial and lingual surfaces is for the purpose of allowing the rubber to extend around and rest against, giving the teeth a more intimate connection with the plate.

The labial surface or gums can be made very thin, without impairing the strength of the teeth, as the rubber will afford a solid and secure base for it to rest upon. Teeth made after this style can be so carved as to need very little or no grinding. There will be no need of pins, nor use for beeswax, in setting up the teeth to be antagonised. Those living close to dental depots can take their model and select just such teeth as are needed. They can be made in blocks of two, three, four or any other number up to a half set. The eight front teeth in a single block, and the rest in two blocks, would make very handsome work.

You will please remember that a similar description and model were sent to Dr. W. B. Franklin in May, 1861.

If teeth were made as above described prior to May, 1861, I am not aware of it; and till made acquainted with the fact, will consider myself the first to make the suggestion.

EASY PLAN OF BRINGING FLASKS TOGETHER.

An item in regard to packing rubber:—I find it most convenient, when I have the rubber to pack, to take the flask or flasks to my kitchen stove, place them in the oven (while cooking is going on), let them dry sufficiently, pack the rubber, previously warmed, by placing on a cloth on the stove, adjust the halves of flask, put on clamp, and tighten slightly, then submerge the flask in boiling water for a short time; remove and bring the parts firmly together, which will be easily and readily accomplished, impressing the rubber into every little nook and corner on the model and around the teeth and pins, if the flask was left in the water a sufficient length of time.

Respectfully, C. B. Myers.

The model referred to above represents a block of eight teeth, with the base a little broader than the ordinary block, and a dove-tailed groove in the base, or that part that rests upon the plate. This is about two lines deep, and two or three wide; the body of the block at the bottom of the groove is perforated by three or four holes, perhaps a line in depth. These measurements, of course, would be regulated by the kind and size of block. This groove and the holes are all filled with rubber, and this constitutes the block attachment. T.

